# Bis(4-amino­pyridinium) sulfate monohydrate

**DOI:** 10.1107/S1600536810030941

**Published:** 2010-08-11

**Authors:** Ching Kheng Quah, Hoong-Kun Fun, Arun M. Isloor, Nishitha Isloor

**Affiliations:** a X-ray Crystallography Unit, School of Physics, Universiti Sains Malaysia, 11800 USM, Penang, Malaysia; b Organic Chemistry Division, Department of Chemistry, National Institute of Technology–Karnataka, Surathkal, Mangalore 575 025, India; c Biotechnology Division, Chemical Engineering Department, National Institute of Technology–Karnataka, Surathkal, Mangalore 575 025, India

## Abstract

The asymmetric unit of the title compound, 2C_5_H_7_N_2_^+^·SO_4_^2−^·H_2_O, contains two 4-amino­pyridinium cations (*A* and *B*), a sulfate dianion and a water mol­ecule. One of the 4-amino­pyridinium cations (*B*) is disordered over two orientations with refined site occupancies of 0.568 (4) and 0.432 (4). The non-H atoms of the 4-amino­pyridinium cations are essentially coplanar, with a maximum deviation of 0.055 (1) Å (in cation *A*), 0.022 (3) Å (for the major component in cation *B*) and 0.009 (3) Å (for the minor component in cation *B*). In the crystal, the sulfate O atoms link the 4-amino­pyridinium cations and water mol­ecules into a three-dimensional network *via* inter­molecular O—H⋯O, N—H⋯O and C—H⋯O hydrogen bonds. The crystal structure is further consolidated by N—H⋯O(water) and C—H⋯O(water) hydrogen bonds.

## Related literature

For general background to and the applications of the title compound, see: Judge & Bever (2006 ▶); Schwid *et al.* (1997 ▶); Strupp *et al.* (2004 ▶); Onoda *et al.* (2001 ▶); Zhang *et al.* (2004 ▶); Pflugrath & Quiocho, (1985 ▶); Jacobson & Quiocho (1988 ▶). For related structures, see: Quah *et al.* (2008*a* ▶,*b* ▶, 2010 ▶). For the stability of the temperature controller used in the data collection, see: Cosier & Glazer (1986 ▶). For bond-length data, see: Allen *et al.* (1987 ▶).
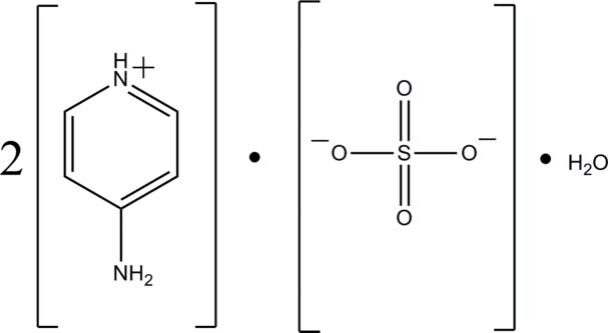


## Experimental

### 

#### Crystal data

2C_5_H_7_N_2_^+^·SO_4_^2−^·H_2_O
*M* *_r_* = 304.33
Triclinic, 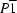

*a* = 6.4434 (1) Å
*b* = 8.4153 (1) Å
*c* = 12.4488 (2) Å
α = 96.365 (1)°
β = 97.534 (1)°
γ = 95.387 (1)°
*V* = 661.02 (2) Å^3^
*Z* = 2
Mo *K*α radiation
μ = 0.27 mm^−1^
*T* = 100 K
0.33 × 0.25 × 0.07 mm

#### Data collection

Bruker SMART APEXII CCD area-detector diffractometer
Absorption correction: multi-scan (*SADABS*; Bruker, 2009 ▶) *T*_min_ = 0.916, *T*_max_ = 0.981
10717 measured reflections
3839 independent reflections
3279 reflections with *I* > 2σ(*I*)
*R*_int_ = 0.027

#### Refinement

*R*[*F* ^2^ > 2σ(*F* ^2^)] = 0.040
*wR*(*F* ^2^) = 0.106
*S* = 1.06
3839 reflections
259 parameters
H atoms treated by a mixture of independent and constrained refinement
Δρ_max_ = 0.59 e Å^−3^
Δρ_min_ = −0.55 e Å^−3^

### 

Data collection: *APEX2* (Bruker, 2009 ▶); cell refinement: *SAINT* (Bruker, 2009 ▶); data reduction: *SAINT*; program(s) used to solve structure: *SHELXTL* (Sheldrick, 2008 ▶); program(s) used to refine structure: *SHELXTL*; molecular graphics: *SHELXTL*; software used to prepare material for publication: *SHELXTL* and *PLATON* (Spek, 2009 ▶).

## Supplementary Material

Crystal structure: contains datablocks global, I. DOI: 10.1107/S1600536810030941/bt5310sup1.cif

Structure factors: contains datablocks I. DOI: 10.1107/S1600536810030941/bt5310Isup2.hkl

Additional supplementary materials:  crystallographic information; 3D view; checkCIF report

## Figures and Tables

**Table 1:** Hydrogen-bond geometry (Å, °)

*D*—H⋯*A*	*D*—H	H⋯*A*	*D*⋯*A*	*D*—H⋯*A*
N2*A*—H2*NA*⋯O1^i^	0.85	2.03	2.822 (3)	156
N2*A*—H1*NA*⋯O1*W*^ii^	0.79	2.07	2.812 (3)	159
N1*A*—H1*AB*⋯O1^iii^	0.86	2.20	2.938 (4)	144
O1*W*—H1*W*1⋯O2^iv^	0.91 (3)	1.88 (3)	2.7952 (19)	176 (3)
O1*W*—H2*W*1⋯O1^v^	0.83 (3)	2.00 (3)	2.8195 (18)	167 (2)
N1—H1*N*1⋯O4^iv^	0.88 (3)	1.85 (2)	2.7102 (17)	165 (2)
N2—H1*N*2⋯O4	0.86 (3)	2.01 (3)	2.8665 (17)	176 (2)
N2—H2*N*2⋯O3^vi^	0.862 (19)	1.96 (2)	2.8118 (17)	167.9 (16)
C1—H1*A*⋯O2^vii^	0.93	2.46	3.3688 (18)	167
C1*A*—H1*AA*⋯O1*W*^viii^	0.93	2.58	3.318 (4)	137
C5*A*—H5*AA*⋯O2^iii^	0.93	2.44	3.228 (7)	143
C4*A*—H4*AA*⋯O3^i^	0.93	2.54	3.362 (6)	147
C5—H5*A*⋯O4^i^	0.93	2.52	3.3360 (18)	146

Symmetry codes: (i) 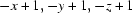; (ii) 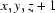; (iii) 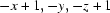; (iv) 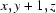; (v) 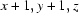; (vi) 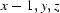; (vii) 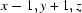; (viii) 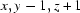.

**Fig. 1.**
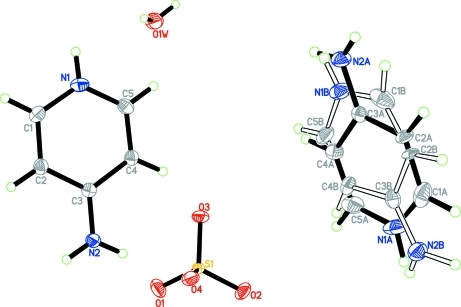
The molecular structure of the title compound showing 50% probability displacement ellipsoids for non-H atoms and the atom-numbering scheme. Both disordered components are shown.

**Fig. 2.**
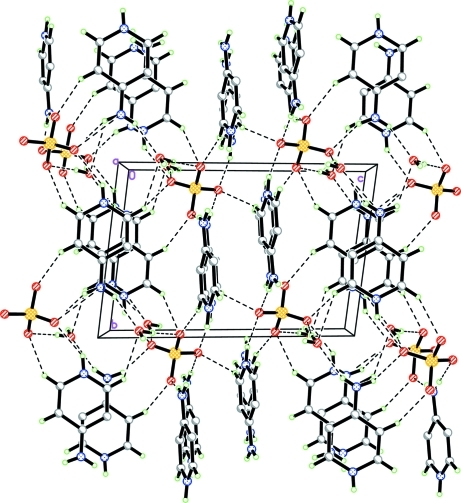
The crystal structure of the title compound viewed along the a axis. H atoms not involved in intermolecular interactions (dashed lines) have been omitted for clarity. Only major disorder component is shown.

## supplementary crystallographic information

### Comment

4-Aminopyridine (fampridine) is clinically used in the treatment of Lambert-Eaton myasthenic syndrome and multiple sclerosis. It prolongs action potentials by blocking potassium channels, thereby increases transmitter release at the neuromuscular junction (Judge & Bever, 2006; Schwid *et al.*, 1997; Strupp *et al.*, 2004). Hydrogen bonding patterns involving sulfate and sulfonate groups in biological systems and metal complexes are also of current interest (Onoda *et al.*, 2001). Benzoic acid and sulfuric acid form a stable hydrogen-bonded complex that favors aerosol formation in the atmosphere (Zhang *et al.*, 2004). In a sulfate-binding protein, the sulfate anion is mainly bonded by seven hydrogen bonds, five of which are from the main chain peptide NH groups (Pflugrath & Quiocho, 1985; Jacobson & Quiocho, 1988). The present study is aimed at understanding the hydrogen bonding network in the title compound (I).

The asymmetric unit of (I) contains two 4-aminopyridinium cations (*A* and *B*), a sulfate anion and a water molecule (Fig 1). The 4-aminopyridinium cation in molecule *B* is disordered over two positions with refined site-occupancies of 0.568 (4) and 0.432 (4). In the 4-aminopyridinium cations, the protonation of atoms N1 (molecule *A*), N1A (major component in molecule *B*) and N1B (minor component in molecule *B*) have lead to slight increase in C–N–C angles to 120.88 (13), 120.9 (4) and 119.7 (5)°, respectively. The non-H atoms of the 4-aminopyridinium cations are essentially co-planar, with a maximum deviation of 0.055 (1) Å for atom N2, 0.022 (3) Å for atom N2A and 0.009 (3) Å for atom N2B. The bond lengths (Allen *et al.*, 1987) and angles are normal and comparable to the related structures (Quah *et al.*, 2008*a*,*b*; 2010). The pyridine ring (N1/C1—C5) in molecule *A* makes dihedral angles of 68.46 (19) and 70.1 (2)°, respectively, with N1A/C1A—C5A and N1B/C1B—C5B rings.

In the crystal packing (Fig. 2), oxygen atoms (O1, O2, O3 and O4) in sulfate anions link the 4-aminopyridinium cations and water molecules into three-dimensional network *via* intermolecular O–H···O, N–H···O and C–H···O hydrogen bonds (Table 1). The crystal structure is further consolidated by N2A–H1NA···O1W and C1A–H1AA···O1W hydrogen bonds.

### Experimental

Few drops of concentrated sulfuric acid were added to a hot methanol solution of 4-aminopyridine (47 mg, Aldrich). The solution was warmed over a water bath for 1 h. The resulting solution was allowed to cool slowly to room temperature. Colourless crystals appeared from the mother liquor after a few days. Yield 60%.

### Refinement

Atoms H1NA, H2NA, H1NB, H2NB, H1AB and H1BA were located in a difference Fourier map and refined using a riding model with N–H = 0.7856 – 0.8900 Å. The remaining O– and N– bound H atoms were located in a difference Fourier map and allowed to refine freely. The rest of the hydrogen atoms were positioned geometrically and refined using a riding model with C–H = 0.93 Å and *U*_iso_(H) = 1.2 *U*_eq_(C). One of the 4-aminopyridinium cations is disordered over two positions with refined site-occupancies of 0.568 (4) and 0.432 (4). The same U^ij^ parameters were used for atom pair C1A/C2A. The highest residual electron density peak is located at 0.72 Å from O1W and the deepest hole is located at 0.68 Å from O1W.

### Crystal data

**Table:**

2C_5_H_7_N_2_^+^·SO_4_^2^^−^·H_2_O	*Z* = 2
*M**_r_* = 304.33	*F*(000) = 320
Triclinic, *P*1	*D*_x_ = 1.529 Mg m^−^^3^
Hall symbol: -P 1	Mo *K*α radiation, λ = 0.71073 Å
*a* = 6.4434 (1) Å	Cell parameters from 3597 reflections
*b* = 8.4153 (1) Å	θ = 3.1–30.1°
*c* = 12.4488 (2) Å	µ = 0.27 mm^−^^1^
α = 96.365 (1)°	*T* = 100 K
β = 97.534 (1)°	Plate, colourless
γ = 95.387 (1)°	0.33 × 0.25 × 0.07 mm
*V* = 661.02 (2) Å^3^	

### Data collection

**Table:**

Bruker SMART APEXII CCD area-detector diffractometer	3839 independent reflections
Radiation source: fine-focus sealed tube	3279 reflections with *I* > 2σ(*I*)
graphite	*R*_int_ = 0.027
φ and ω scans	θ_max_ = 30.0°, θ_min_ = 1.7°
Absorption correction: multi-scan (*SADABS*; Bruker, 2009)	*h* = −9→9
*T*_min_ = 0.916, *T*_max_ = 0.981	*k* = −11→11
10717 measured reflections	*l* = −17→17

### Refinement

**Table:**

Refinement on *F*^2^	Primary atom site location: structure-invariant direct methods
Least-squares matrix: full	Secondary atom site location: difference Fourier map
*R*[*F*^2^ > 2σ(*F*^2^)] = 0.040	Hydrogen site location: inferred from neighbouring sites
*wR*(*F*^2^) = 0.106	H atoms treated by a mixture of independent and constrained refinement
*S* = 1.06	*w* = 1/[σ^2^(*F*_o_^2^) + (0.0441*P*)^2^ + 0.4122*P*] where *P* = (*F*_o_^2^ + 2*F*_c_^2^)/3
3839 reflections	(Δ/σ)_max_ < 0.001
259 parameters	Δρ_max_ = 0.59 e Å^−^^3^
0 restraints	Δρ_min_ = −0.55 e Å^−^^3^

### Special details

**Table:**

Experimental. The crystal was placed in the cold stream of an Oxford Cyrosystems Cobra open-flow nitrogen cryostat (Cosier & Glazer, 1986) operating at 100.0 (1) K.
Geometry. All e.s.d.'s (except the e.s.d. in the dihedral angle between two l.s. planes) are estimated using the full covariance matrix. The cell e.s.d.'s are taken into account individually in the estimation of e.s.d.'s in distances, angles and torsion angles; correlations between e.s.d.'s in cell parameters are only used when they are defined by crystal symmetry. An approximate (isotropic) treatment of cell e.s.d.'s is used for estimating e.s.d.'s involving l.s. planes.
Refinement. Refinement of *F*^2^ against ALL reflections. The weighted *R*-factor *wR* and goodness of fit *S* are based on *F*^2^, conventional *R*-factors *R* are based on *F*, with *F* set to zero for negative *F*^2^. The threshold expression of *F*^2^ > σ(*F*^2^) is used only for calculating *R*-factors(gt) *etc*. and is not relevant to the choice of reflections for refinement. *R*-factors based on *F*^2^ are statistically about twice as large as those based on *F*, and *R*- factors based on ALL data will be even larger.

### Fractional atomic coordinates and isotropic or equivalent isotropic displacement parameters (Å^2^)

**Table:**

	*x*	*y*	*z*	*U*_iso_*/*U*_eq_	Occ. (<1)
S1	0.41695 (5)	0.12500 (4)	0.29301 (3)	0.01520 (10)	
O4	0.28974 (17)	0.13054 (13)	0.38487 (9)	0.0210 (2)	
O3	0.52927 (17)	0.28476 (13)	0.29263 (10)	0.0217 (2)	
O2	0.56565 (18)	0.00390 (14)	0.30613 (10)	0.0256 (3)	
O1	0.27120 (18)	0.08095 (15)	0.18901 (10)	0.0277 (3)	
N2	−0.0310 (2)	0.34101 (15)	0.35023 (11)	0.0172 (2)	
C5	0.2971 (2)	0.72142 (18)	0.42897 (12)	0.0182 (3)	
H5A	0.4373	0.7607	0.4527	0.022*	
N1	0.1519 (2)	0.82613 (16)	0.41396 (11)	0.0192 (3)	
C3	0.0277 (2)	0.49838 (17)	0.37303 (11)	0.0144 (3)	
C2	−0.1204 (2)	0.61239 (18)	0.36079 (12)	0.0168 (3)	
H2A	−0.2625	0.5775	0.3392	0.020*	
O1W	0.8386 (2)	0.96841 (16)	0.15025 (14)	0.0355 (3)	
C4	0.2413 (2)	0.55943 (17)	0.40997 (11)	0.0160 (3)	
H4A	0.3428	0.4891	0.4212	0.019*	
C1	−0.0540 (2)	0.77280 (18)	0.38069 (12)	0.0189 (3)	
H1A	−0.1514	0.8467	0.3713	0.023*	
C2A	0.7722 (12)	0.4735 (7)	1.0588 (7)	0.0238 (7)	0.568 (4)
H2AA	0.8019	0.5311	1.1282	0.029*	0.568 (4)
C1A	0.7613 (4)	0.3094 (4)	1.0482 (3)	0.0238 (7)	0.568 (4)
H1AA	0.7854	0.2567	1.1097	0.029*	0.568 (4)
C5A	0.6790 (11)	0.2982 (7)	0.8591 (6)	0.0237 (11)	0.568 (4)
H5AA	0.6461	0.2370	0.7910	0.028*	0.568 (4)
C4A	0.6891 (9)	0.4594 (7)	0.8661 (5)	0.0175 (10)	0.568 (4)
H4AA	0.6624	0.5082	0.8030	0.021*	0.568 (4)
N2A	0.7527 (4)	0.7162 (3)	0.9771 (2)	0.0185 (6)	0.568 (4)
H2NA	0.7286	0.7515	0.9160	0.028*	0.568 (4)
H1NA	0.7770	0.7672	1.0350	0.028*	0.568 (4)
C3A	0.7401 (4)	0.5559 (4)	0.9691 (2)	0.0134 (6)	0.568 (4)
N1A	0.7155 (4)	0.2243 (4)	0.9482 (3)	0.0263 (7)	0.568 (4)
H1AB	0.7039	0.1217	0.9350	0.032*	0.568 (4)
N2B	0.7174 (5)	0.1525 (5)	0.9860 (3)	0.0219 (8)	0.432 (4)
H1NB	0.7542	0.1178	1.0500	0.033*	0.432 (4)
H2NB	0.7106	0.0855	0.9312	0.033*	0.432 (4)
C1B	0.7723 (6)	0.5882 (5)	1.0409 (4)	0.0270 (10)	0.432 (4)
H1BB	0.7821	0.6760	1.0868	0.040*	0.432 (4)
N1B	0.7377 (5)	0.6283 (6)	0.9395 (4)	0.0257 (9)	0.432 (4)
H1BA	0.7421	0.7279	0.9294	0.038*	0.432 (4)
C3B	0.7256 (5)	0.3062 (6)	0.9719 (3)	0.0173 (8)	0.432 (4)
C4B	0.6880 (12)	0.3543 (9)	0.8640 (6)	0.0168 (13)	0.432 (4)
H4BA	0.6584	0.2771	0.8030	0.020*	0.432 (4)
C5B	0.6959 (13)	0.5103 (9)	0.8524 (7)	0.0208 (13)	0.432 (4)
H5BA	0.6721	0.5400	0.7824	0.025*	0.432 (4)
C2B	0.7688 (12)	0.4344 (6)	1.0631 (6)	0.0153 (12)	0.432 (4)
H2BA	0.7932	0.4112	1.1347	0.018*	0.432 (4)
H1W1	0.750 (5)	0.985 (4)	0.201 (3)	0.067 (9)*	
H2W1	0.963 (5)	1.006 (3)	0.172 (2)	0.054 (8)*	
H1N1	0.193 (3)	0.929 (3)	0.4163 (18)	0.033 (6)*	
H1N2	0.061 (4)	0.275 (3)	0.3623 (17)	0.031 (6)*	
H2N2	−0.163 (3)	0.309 (2)	0.3310 (16)	0.023 (5)*	

### Atomic displacement parameters (Å^2^)

**Table:**

	*U*^11^	*U*^22^	*U*^33^	*U*^12^	*U*^13^	*U*^23^
S1	0.01105 (16)	0.01034 (16)	0.02362 (18)	0.00082 (11)	0.00082 (12)	0.00180 (12)
O4	0.0194 (5)	0.0152 (5)	0.0305 (6)	0.0027 (4)	0.0077 (4)	0.0058 (4)
O3	0.0194 (5)	0.0141 (5)	0.0311 (6)	−0.0032 (4)	0.0040 (4)	0.0044 (4)
O2	0.0208 (6)	0.0197 (5)	0.0401 (7)	0.0100 (4)	0.0077 (5)	0.0098 (5)
O1	0.0191 (5)	0.0286 (6)	0.0303 (6)	0.0024 (5)	−0.0056 (5)	−0.0071 (5)
N2	0.0142 (6)	0.0140 (6)	0.0232 (6)	0.0016 (5)	0.0031 (5)	0.0004 (5)
C5	0.0158 (6)	0.0202 (7)	0.0181 (6)	−0.0016 (5)	0.0036 (5)	0.0021 (5)
N1	0.0226 (6)	0.0137 (6)	0.0217 (6)	−0.0008 (5)	0.0062 (5)	0.0027 (5)
C3	0.0158 (6)	0.0156 (6)	0.0123 (6)	0.0011 (5)	0.0036 (5)	0.0026 (5)
C2	0.0152 (6)	0.0182 (7)	0.0176 (6)	0.0024 (5)	0.0028 (5)	0.0032 (5)
O1W	0.0192 (6)	0.0249 (6)	0.0622 (10)	0.0008 (5)	0.0094 (6)	0.0017 (6)
C4	0.0145 (6)	0.0175 (7)	0.0163 (6)	0.0025 (5)	0.0024 (5)	0.0029 (5)
C1	0.0206 (7)	0.0169 (7)	0.0210 (7)	0.0049 (5)	0.0052 (5)	0.0048 (5)
C2A	0.0165 (11)	0.0299 (17)	0.0289 (14)	0.0066 (10)	0.0065 (9)	0.0130 (11)
C1A	0.0165 (11)	0.0299 (17)	0.0289 (14)	0.0066 (10)	0.0065 (9)	0.0130 (11)
C5A	0.0209 (17)	0.016 (3)	0.0340 (19)	0.003 (2)	0.0088 (13)	−0.005 (2)
C4A	0.0193 (15)	0.015 (3)	0.0171 (19)	0.001 (2)	0.0023 (12)	−0.0004 (19)
N2A	0.0209 (11)	0.0122 (13)	0.0214 (11)	0.0026 (8)	0.0036 (8)	−0.0034 (9)
C3A	0.0089 (11)	0.0152 (14)	0.0161 (15)	0.0024 (9)	0.0029 (9)	0.0000 (11)
N1A	0.0228 (13)	0.0104 (15)	0.0475 (19)	0.0020 (11)	0.0117 (12)	0.0029 (15)
N2B	0.0266 (17)	0.0202 (18)	0.0188 (15)	0.0024 (13)	0.0015 (12)	0.0037 (13)
C1B	0.0173 (18)	0.025 (2)	0.036 (3)	−0.0006 (14)	0.0071 (16)	−0.0072 (18)
N1B	0.0165 (16)	0.015 (2)	0.047 (2)	0.0037 (13)	0.0092 (14)	0.004 (2)
C3B	0.0114 (15)	0.020 (2)	0.020 (2)	0.0028 (14)	0.0025 (12)	0.0010 (16)
C4B	0.019 (2)	0.018 (4)	0.016 (2)	0.005 (3)	0.0026 (15)	0.007 (3)
C5B	0.018 (2)	0.017 (4)	0.030 (3)	0.007 (3)	0.0061 (17)	0.008 (2)
C2B	0.0112 (16)	0.029 (3)	0.0062 (16)	0.004 (2)	0.0031 (12)	0.002 (2)

### Geometric parameters (Å, °)

**Table:**

S1—O3	1.4666 (11)	C5A—N1A	1.335 (9)
S1—O2	1.4706 (11)	C5A—C4A	1.345 (5)
S1—O1	1.4849 (12)	C5A—H5AA	0.9300
S1—O4	1.4909 (12)	C4A—C3A	1.422 (6)
N2—C3	1.3311 (18)	C4A—H4AA	0.9300
N2—H1N2	0.86 (2)	N2A—C3A	1.335 (4)
N2—H2N2	0.86 (2)	N2A—H2NA	0.8477
C5—N1	1.354 (2)	N2A—H1NA	0.7856
C5—C4	1.363 (2)	N1A—H1AB	0.8555
C5—H5A	0.9300	N2B—C3B	1.321 (6)
N1—C1	1.355 (2)	N2B—H1NB	0.8900
N1—H1N1	0.88 (2)	N2B—H2NB	0.8295
C3—C4	1.4208 (19)	C1B—N1B	1.339 (6)
C3—C2	1.421 (2)	C1B—C2B	1.352 (6)
C2—C1	1.362 (2)	C1B—H1BB	0.8750
C2—H2A	0.9300	N1B—C5B	1.367 (11)
O1W—H1W1	0.91 (3)	N1B—H2NA	1.1125
O1W—H2W1	0.83 (3)	N1B—H1BA	0.8600
C4—H4A	0.9300	C3B—C4B	1.443 (8)
C1—H1A	0.9300	C3B—C2B	1.456 (8)
C2A—C1A	1.366 (6)	C4B—C5B	1.334 (7)
C2A—C3A	1.381 (8)	C4B—H4BA	0.9300
C2A—H2AA	0.9300	C5B—H5BA	0.9300
C1A—N1A	1.347 (5)	C2B—H2BA	0.9300
C1A—H1AA	0.9300		
			
O3—S1—O2	110.89 (7)	H1NA—N2A—H1BB	46.1
O3—S1—O1	109.12 (7)	C3A—N2A—H1BA	102.2
O2—S1—O1	109.99 (7)	H1NA—N2A—H1BA	138.1
O3—S1—O4	109.09 (7)	H1BB—N2A—H1BA	175.5
O2—S1—O4	109.35 (7)	N2A—C3A—C2A	122.9 (4)
O1—S1—O4	108.35 (7)	N2A—C3A—C4A	121.2 (4)
C3—N2—H1N2	119.4 (15)	C2A—C3A—C4A	115.9 (4)
C3—N2—H2N2	118.3 (13)	C2A—C3A—H1BA	145.0
H1N2—N2—H2N2	121.9 (19)	C4A—C3A—H1BA	99.1
N1—C5—C4	121.14 (13)	C5A—N1A—C1A	120.9 (4)
N1—C5—H5A	119.4	C5A—N1A—H1AB	113.9
C4—C5—H5A	119.4	C1A—N1A—H1AB	125.2
C5—N1—C1	120.88 (13)	C5A—N1A—H2NB	114.7
C5—N1—H1N1	119.8 (15)	C1A—N1A—H2NB	124.4
C1—N1—H1N1	118.7 (14)	C3B—N2B—H1AB	98.6
N2—C3—C4	121.37 (13)	C3B—N2B—H1NB	123.7
N2—C3—C2	121.41 (13)	H1AB—N2B—H1NB	136.0
C4—C3—C2	117.21 (13)	C3B—N2B—H2NB	117.9
C1—C2—H2A	120.0	H1NB—N2B—H2NB	116.1
C3—C2—H2A	120.0	N1B—C1B—C2B	123.4 (5)
H1W1—O1W—H2W1	114 (3)	N1B—C1B—H1NA	65.4
C5—C4—C3	119.81 (13)	C2B—C1B—H1NA	171.1
C5—C4—H4A	120.1	N1B—C1B—H1BB	108.0
C3—C4—H4A	120.1	C2B—C1B—H1BB	128.3
N1—C1—C2	120.94 (14)	C1B—N1B—C5B	119.7 (5)
N1—C1—H1A	119.5	C1B—N1B—H2NA	126.9
C2—C1—H1A	119.5	C5B—N1B—H2NA	113.3
C1A—C2A—C3A	121.7 (7)	C1B—N1B—H1BA	120.0
C1A—C2A—H2AA	119.1	C5B—N1B—H1BA	120.3
C3A—C2A—H2AA	119.1	N2B—C3B—C4B	120.9 (5)
N1A—C1A—C2A	119.7 (5)	N2B—C3B—C2B	122.3 (4)
N1A—C1A—H1AA	120.1	C4B—C3B—C2B	116.8 (5)
C2A—C1A—H1AA	120.1	C4B—C3B—H1AB	96.6
N1A—C5A—C4A	121.2 (6)	C2B—C3B—H1AB	146.6
N1A—C5A—H5AA	119.4	C5B—C4B—C3B	119.5 (6)
C4A—C5A—H5AA	119.4	C5B—C4B—H4BA	120.2
C5A—C4A—C3A	120.6 (5)	C3B—C4B—H4BA	120.2
C5A—C4A—H4AA	119.7	C4B—C5B—N1B	122.4 (7)
C3A—C4A—H4AA	119.7	C4B—C5B—H5BA	118.8
C3A—N2A—H2NA	113.5	N1B—C5B—H5BA	118.8
C3A—N2A—H1NA	119.5	C1B—C2B—C3B	118.2 (5)
H2NA—N2A—H1NA	127.0	C1B—C2B—H2BA	120.9
C3A—N2A—H1BB	73.5	C3B—C2B—H2BA	120.9
H2NA—N2A—H1BB	172.0		
			
C4—C5—N1—C1	0.7 (2)	C5A—C4A—C3A—N2A	179.3 (4)
N2—C3—C2—C1	−177.42 (14)	C5A—C4A—C3A—C2A	−1.6 (7)
C4—C3—C2—C1	2.2 (2)	C4A—C5A—N1A—C1A	0.7 (7)
N1—C5—C4—C3	0.4 (2)	C2A—C1A—N1A—C5A	−0.5 (7)
N2—C3—C4—C5	177.75 (13)	C2B—C1B—N1B—C5B	0.1 (8)
C2—C3—C4—C5	−1.9 (2)	N2B—C3B—C4B—C5B	179.5 (5)
C5—N1—C1—C2	−0.4 (2)	C2B—C3B—C4B—C5B	0.6 (8)
C3—C2—C1—N1	−1.1 (2)	C3B—C4B—C5B—N1B	−0.3 (10)
C3A—C2A—C1A—N1A	−0.9 (8)	C1B—N1B—C5B—C4B	0.0 (9)
N1A—C5A—C4A—C3A	0.3 (8)	N1B—C1B—C2B—C3B	0.2 (8)
C1A—C2A—C3A—N2A	−179.1 (4)	N2B—C3B—C2B—C1B	−179.4 (5)
C1A—C2A—C3A—C4A	1.8 (8)	C4B—C3B—C2B—C1B	−0.5 (8)

### Hydrogen-bond geometry (Å, °)

**Table:**

*D*—H···*A*	*D*—H	H···*A*	*D*···*A*	*D*—H···*A*
N2A—H2NA···O1^i^	0.85	2.03	2.822 (3)	156.
N2A—H1NA···O1W^ii^	0.79	2.07	2.812 (3)	159.
N1A—H1AB···O1^iii^	0.86	2.20	2.938 (4)	144.
O1W—H1W1···O2^iv^	0.91 (3)	1.88 (3)	2.7952 (19)	176 (3)
O1W—H2W1···O1^v^	0.83 (3)	2.00 (3)	2.8195 (18)	167 (2)
N1—H1N1···O4^iv^	0.88 (3)	1.85 (2)	2.7102 (17)	165 (2)
N2—H1N2···O4	0.86 (3)	2.01 (3)	2.8665 (17)	176 (2)
N2—H2N2···O3^vi^	0.862 (19)	1.96 (2)	2.8118 (17)	167.9 (16)
C1—H1A···O2^vii^	0.93	2.46	3.3688 (18)	167.
C1A—H1AA···O1W^viii^	0.93	2.58	3.318 (4)	137.
C5A—H5AA···O2^iii^	0.93	2.44	3.228 (7)	143.
C4A—H4AA···O3^i^	0.93	2.54	3.362 (6)	147.
C5—H5A···O4^i^	0.93	2.52	3.3360 (18)	146.

Symmetry codes: (i) −*x*+1, −*y*+1, −*z*+1; (ii) *x*, *y*, *z*+1; (iii) −*x*+1, −*y*, −*z*+1; (iv) *x*, *y*+1, *z*; (v) *x*+1, *y*+1, *z*; (vi) *x*−1, *y*, *z*; (vii) *x*−1, *y*+1, *z*; (viii) *x*, *y*−1, *z*+1.
